# Investigation of anti-osteoporosis mechanisms of *Rehmanniae Radix Preparata* based on network pharmacology and experimental verification

**DOI:** 10.1186/s13018-021-02751-5

**Published:** 2021-10-14

**Authors:** Li Ou, Wenqian Kang, Ziyi Liang, Feng Gao, Taiwei Dong, Peifeng Wei, Min Li

**Affiliations:** College of Pharmacy, Shaanxi University of Chinese Medicine, Xianyang, 712046 China

**Keywords:** *Rehmanniae Radix Preparata*, Network pharmacology, Mechanism, Osteoporosis, Bone

## Abstract

**Background:**

*Rehmanniae Radix Preparata* (RRP) can effectively improve the symptoms of osteoporosis, but its molecular mechanism for treating osteoporosis is still unclear. The objective of this study is to investigate the anti-osteoporosis mechanisms of RRP through network pharmacology.

**Methods:**

The overlapping targets of RRP and osteoporosis were screened out using online platforms. A visual network diagram of PPI was constructed and analyzed by Cytoscape 3.7.2 software. Molecular docking was used to evaluate the binding activity of ligands and receptors, and some key genes were verified through pharmacological experiments.

**Results:**

According to topological analysis results, AKT1, MAPK1, ESR1, and SRC are critical genes for RRP to treat osteoporosis, and they have high binding activity with stigmasterol and sitosterol. The main signal pathways of RRP in the treatment of osteoporosis, including the estrogen signaling pathway, HIF-1 signal pathway, MAPK signal pathway, PI3K-Akt signal pathway. Results of animal experiments showed that RRP could significantly increase the expression levels of Akt1, MAPK1, ESR1, and SRC1 mRNA in bone tissue to increase bone density.

**Conclusion:**

This study explained the coordination between multiple components and multiple targets of RRP in the treatment of osteoporosis and provided new ideas for its clinical application and experimental research.

## Introduction

Osteoporosis is a common bone metabolism disease in middle-aged and older adults. It often leads to bone deformities and fractures and seriously affects people's health and quality of life [1, 2]. With the aging of the global population, its incidence is increasing year by year, so there is an urgent need to explore effective treatment methods [3, 4]. At present, the treatment of osteoporosis is mainly through applying three types of drugs: bone formation promoters, bone resorption inhibitors, and bone mineral agents to improve patients' clinical symptoms, but these treatment methods have certain limitations [5, 6]. *Rehmanniae Radix Preparata* (RRP) is a Chinese herbal medicine with a long history of treating osteoporosis [7, 8]. Its main chemical components include sterol, styrene glycosides, amino acids, and carbohydrates, reducing bone loss and slowing down aging [9, 10]. However, the material basis and molecular mechanism of RRP in the treatment of osteoporosis are still unclear.

Based on systems biology and bioinformatics, network pharmacology explores the interaction between biomolecules and targets in the body to effectively predict the efficacy and mechanism of drugs [11]. This study integrated information such as active ingredients, drug targets, and disease targets through network pharmacological methods to explore the material basis and mechanism of RRP in the treatment of osteoporosis.

**Fig. 1:**
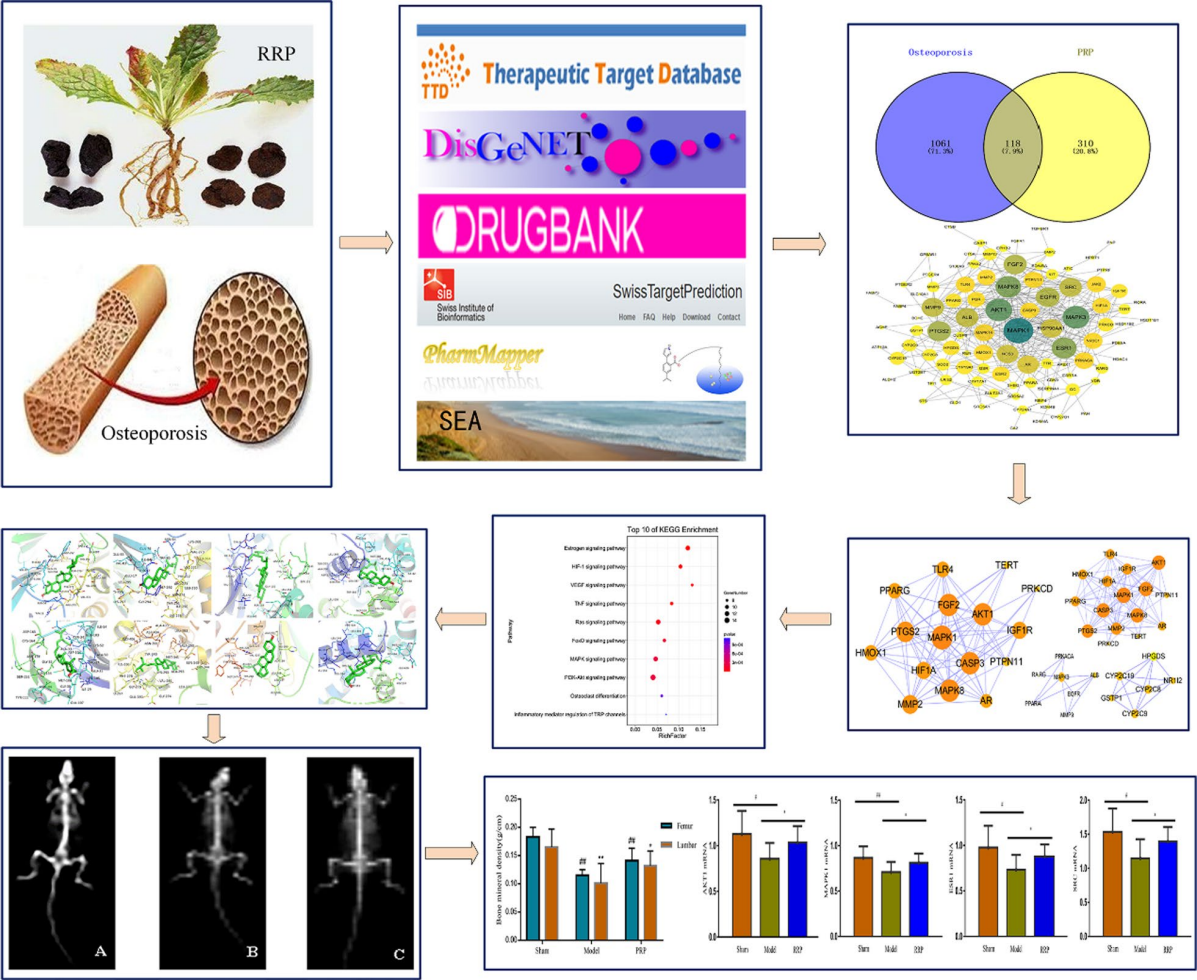
Network pharmacology research flowchart

This study explained the coordination between multiple components and multiple targets of RRP in the treatment of osteoporosis. It provided new ideas for its clinical application and experimental research. First, overlapping targets of RRP and osteoporosis were screened out using online platforms. Next, a visual network diagram of PPI was constructed and analyzed by Cytoscape 3.7.2 software. Finally, molecular docking was used to evaluate the binding activity of ligands and receptors, and some key genes were verified through pharmacological experiments. The research flowchart is shown in Fig. 1.

## Methods

### Screening of anti-osteoporosis targets of RRP

Osteoporosis-related targets were collected from online-accessible databases of DisGeNET (https://www.disgenet.org/), TTD (http://db.idrblab.net/ttd/) and Drukbank (https://www.drugbank.ca/) [12–14]. In addition, we used three online platforms: SEA (http://sea.bkslab.org), PharmMapper (http://www.lilab-ecust.cn/pharmmapper/), and SwissTargetPrediction (http://www.swisstargetprediction.ch/) to search for the target of RRP, and used UniProt database (https://www.Uniprot.org/) to standardize the gene ID [15–17]. Furthermore, all overlapping targets of RRP and osteoporosis were assayed by Venn diagrams to identify the targets for RRP-treated osteoporosis.

### Construction and analysis of protein interaction network

The overlapping targets of RRP and osteoporosis were imported into STRING (https://string-db.org/) to obtain the protein–protein interaction (PPI) [18]. Then we used Cytoscape 3.7.2 software to construct a visual network diagram of PPI and further identified the targets for RRP-treated osteoporosis using cluster analysis [19].

### GO and pathway enrichment analysis for key targets

The key genes were imported into several online biological information databases such as DAVID (version: 6.8) and STRING (version: 11.0), and GO, and KEGG pathway enrichment analyses were performed [20, 21].

### Molecular docking of RRP and key targets

The 3D structure of the target protein was downloaded from PDB (https://www.rcsb.org/), and the water molecules and small molecule ligands of the target protein were removed using Pymol software [22]. Then we used AutoDock Tools software to prepare the hydrogenated protein and calculate the docking score.

### Establishment of the experimental model of osteoporosis

Female SD rats weighing 200 ± 20 g were randomly divided into three groups: sham operation group, model group, and RRP group, with ten rats in each group. The experimental animals were purchased from Sichuan Chengdu Dashuo Experimental Animal Co., Ltd. (Chengdu, China), and the license number is SCXK 2019-028. Rats were kept in a well-ventilated environment with a room temperature of 22–25 °C and relative humidity of 50–60%. Animal experiments were carried out following the principles of the Care and Use of Laboratory Animal.

The rats in the model and RRP groups underwent ovariectomy, while the ovaries in the sham operation group were not removed. After the operation, the vaginal secretions of the rats were collected, and the keratinized epithelial cells were not observed as a critical indicator of successful ovariectomy. Rats in the sham operation group and model group were intragastrically administered with distilled water. Rats in the RRP group were intragastrically administered with a dose of 5.4 g/kg of RRP daily. The rats were dissected, and their femurs were taken after 16 weeks.

### Bone density examination

The rats were anesthetized by intraperitoneal injection of 3% sodium pentobarbital (1 ml/kg), and the right femur and the third lumbar vertebra were peeled off. A dual-energy X-ray bone densitometer (Lunar, USA) was used to detect the bone mineral density of rats' femur and lumbar spine.

### Validation of key targets through qRT-PCR

Four key targets were verified by real-time quantitative reverse transcription (RT-PCR). Primers were designed and synthesized by the solid-phase phosphoramidite triester method, and the sequence of primer was as follows: AKT1 forward primer: 5′-GGCCCAGATGATCACCATCAC-3′; AKT1 reverse primer: 5′-CTATCGTC CAGCGCAGTCCA-3′; MAPK1 forward primer: 5′- TTGCTGAAGCACCATTCAAG-3′; MAPK1 reverse primer: 5′-ACGGCTCAAAGGAGTCAAGA-3′; ESR1 forward primer: 5′-CCAACCAGTGCACCATTGAT-3′; ESR1 reverse primer: 5′-TTTGATCATGAGCGGGCTTG-3-3′; SRC1 forward primer: 5′-CAACCAGCAAAGGCTGAGTCCA-3′; SRC1 reverse primer: 5′-AGTACCTCCTGAGGGGTTAGAG-3′. RNA of rats left femur were extracted with EasyPureTM RNA Kit (TransGen, China). TransScript First-strand cDNA Synthesis SuperMix Kit (TransGen, China) was used for reverse transcription reaction. The program used consisted of a pre-denaturation step of 95 °C for 3 min, 40 cyclings of denaturation 94 °C for 15 s, annealing temperature 50 °C for 30 s, extension 72 °C for 1 min, and a final extension step of 72 °C for 5 min. The gene expression data were analyzed by using the 2^−ΔΔCT^ method.

### Statistical analysis

All statistical analyses were performed using SPSS19.0 software. All data were expressed as mean ± standard deviation ($\overline{x}$ ± s). One-way analysis of variance was used to analyze the data from multiple groups. *P* values of 0.05 or less were regarded as statistically significant.

## Results

### Active ingredients and targets of RRP in the treatment of osteoporosis

A total of 76 active ingredients of RRP were searched, and two active ingredients were screened based on oral bioavailability (OB) > 30% and drug-likeness (DL) > 0.18, including β-sitosterol (MOL000359) and stigmasterol (MOL000449). It was reported in the literature that 5-HMF could promote osteoblast production and might be one of the components of RRP in the treatment of osteoporosis. Therefore, although the DL value of 5-HMF (MOL000748) did not meet the standard, it was also included as an active ingredient (Table 1). We searched three online Platforms with the keyword “Osteoporosis” and identified 1179 osteoporosis-related targets. And we also obtained 428 RRP targets after removing duplicates. Finally, we found that there were a total of 118 overlapping targets for RRP and osteoporosis.

**Table 1:**
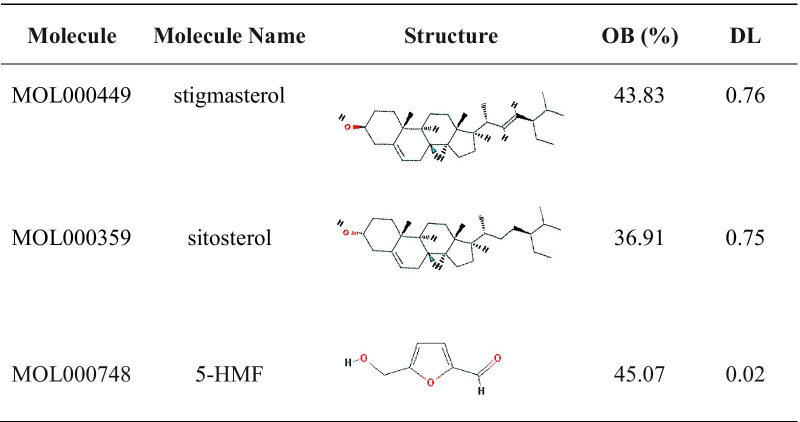
Active ingredients of RRP

RRP: *Rehmanniae Radix Preparata*; OB: oral bioavailability; DL: drug-likeness

### Network construction and analysis

**Fig. 2:**
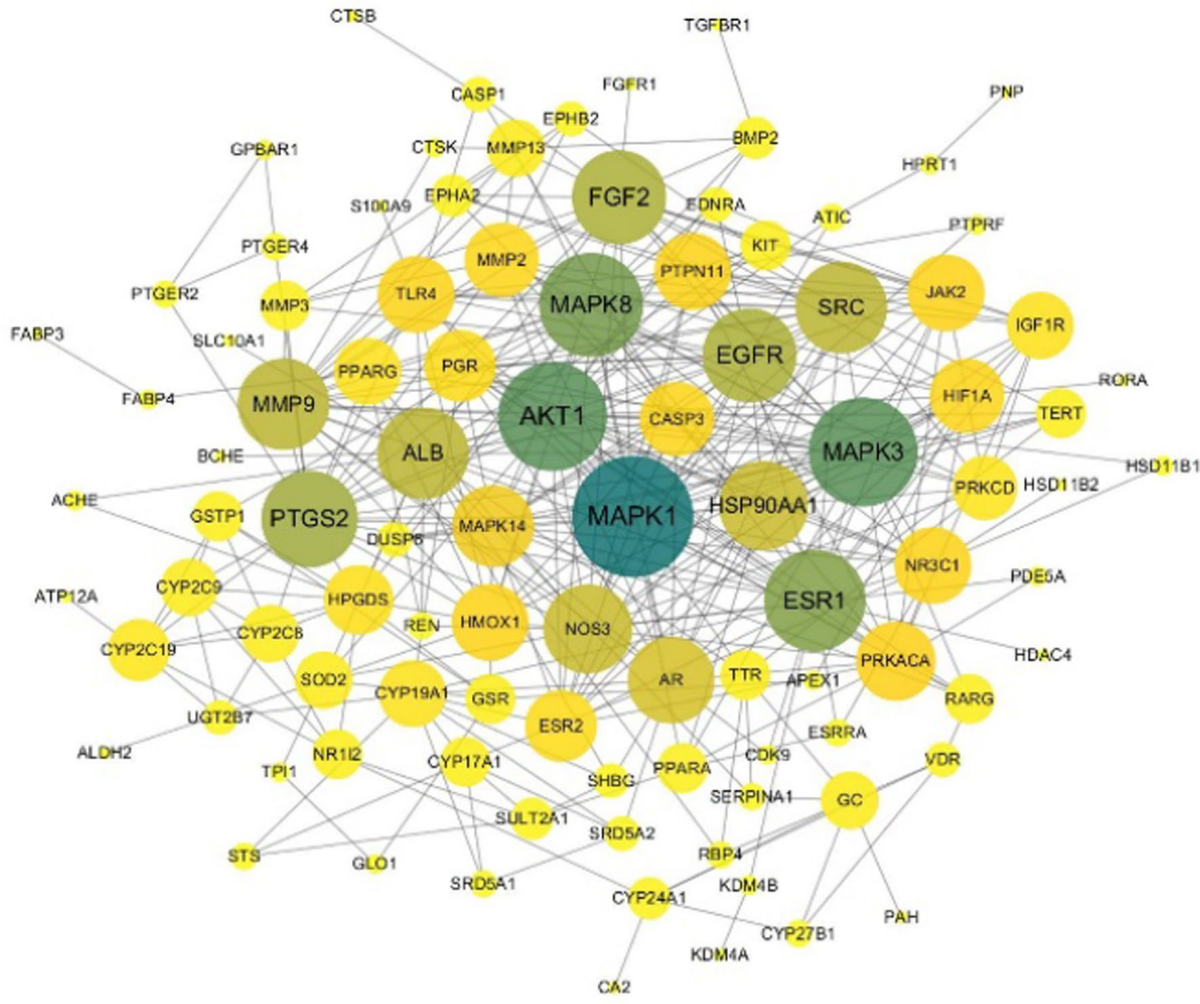
PPI network. PPI: protein–protein interaction

After the overlapping targets were uploaded to STRING (at 70% confidence), the PPI network with 98 nodes and 378 edges was constructed using Cytoscape 3.7.2 software (Fig. 2). In the generated network, nodes represented targets, and edges represented the interaction between targets. We used the Cytohub plug-in to analyze the network topology properties. The degree value of the node reflected the importance of the node in the network. In the PPI network, the node color changed from yellow to green reflected the degree value changed from low to high. The top 10 genes were MAPK1, MAPK3, AKT1, MAPK8, ESR1, PTGS2, EGFR, FGF2, SRC, MMP9. Their degree values were more than twofold the median degree of all nodes in the network [23].

**Fig. 3:**
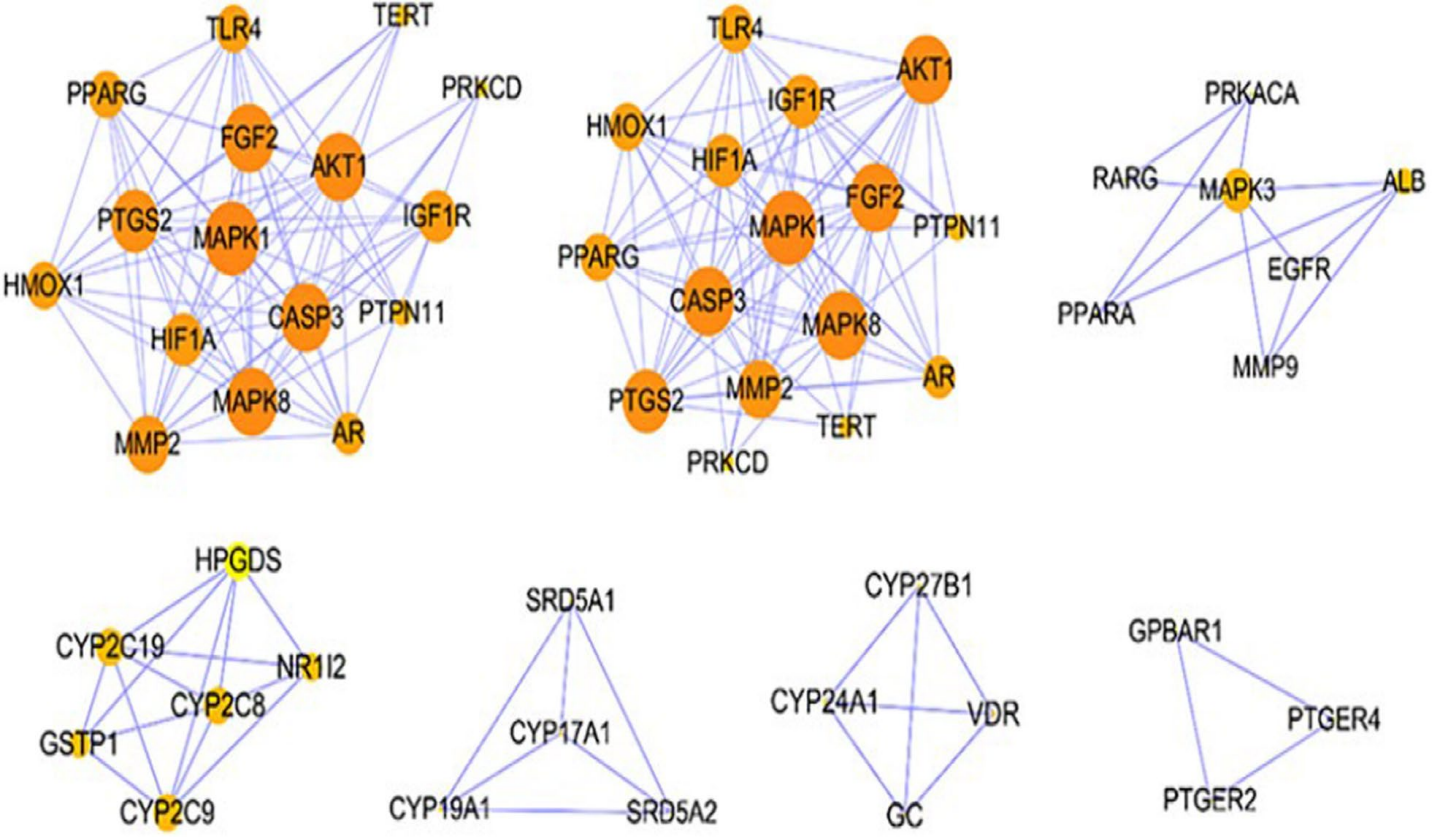
Densely linked modules included in target network of RRP

The MCODE plug-in was used to decompose the PPI network, and seven closely connected sub-modules in the network were identified, including two 16-core (the connectivity of each node in the module is at least 16), one 7-core, one 6-core, two 4-core, and one 3-core (Fig. 3). This sub-module reflected the closely related protein's interaction that completed specific molecular functions. The genes in these sub-modules were closely related to the following molecular functions: enzyme binding, phosphotransferase activity, signaling receptor binding, protein kinase binding, protein tyrosine kinase activity, ion binding, steroid hormone receptor activity, phosphatidylinositol-4,5-bisphosphate 3-kinase activity, heme binding, G protein-coupled receptor activity. And these genes were involved in many critical biological processes related to osteoporosis, such as regulation of cell population proliferation, positive regulation of nitrogen compound metabolic process, activation of protein kinase activity, positive regulation of reactive oxygen species metabolic process, regulation of phosphorylation, steroid metabolic process, vitamin D metabolic process, bone development, regulation of protein binding and G protein-coupled receptor signaling pathway.

### Enrichment analysis of key targets

**Fig. 4:**
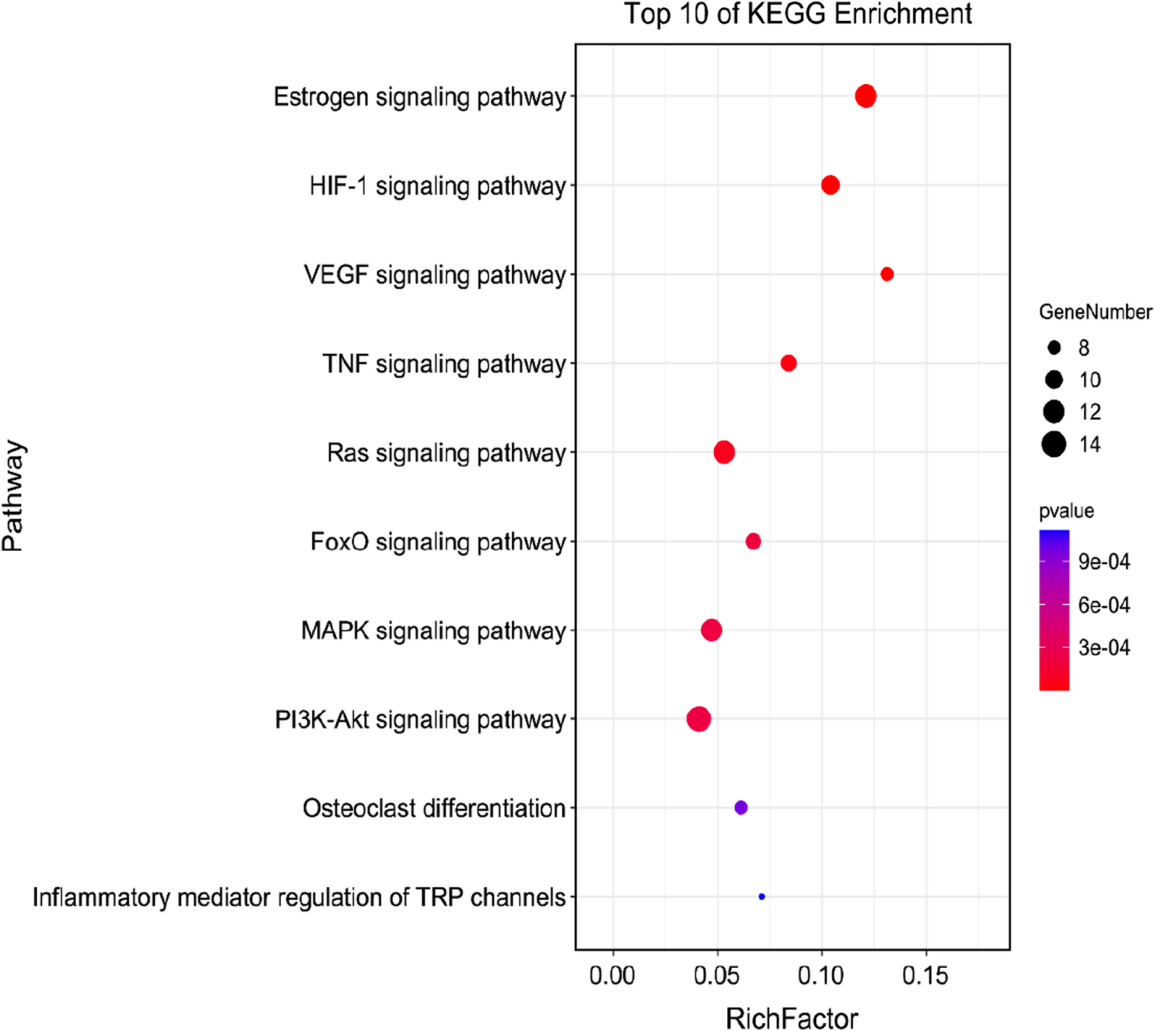
Bubble diagram of top 10 KEGG enrichment pathways

In the results of the enrichment of KEGG pathways, the pathways of fundamental biological processes were screened with a false discovery rate (FDR) less than 0.01, and an enriched cluster containing 162 pathways was obtained (enrichment score = 2.12). According to the FDR value of these pathways, ten pathways related to osteoporosis were screened out, including the estrogen signaling pathway, HIF-1 signaling pathway, VEGF signaling pathway, TNF signaling pathway, Ras signaling pathway, FoxO signaling pathway, MAPK signaling pathway, PI3K-Akt signaling pathway, Osteoclast differentiation and Inflammatory mediator regulation of TRP channels (Table 2). Then we classified and visualized the pathways based on the number of key genes in these pathways (Fig. 4). The classification of these pathways belongs to the endocrine system, signal transduction, development and regeneration, and sensory system, which were the critical target pathways of RRP to interfere with the biological process of osteoporosis.

**Table 2:** KEGG signaling pathways regulated by important targets

Category	Pathway	Number of genes	Mapped targets	FDR
Endocrine system	Estrogen signaling pathway	99	12	8.91 × 10^–7^
Signal transduction	HIF-1 signaling pathway	96	10	4.82 × 10^–5^
Signal transduction	VEGF signaling pathway signaling pathway	61	8	1.33 × 10^–4^
Signal transduction	TNF signaling pathway	107	9	4.21 × 10^–4^
Signal transduction	Ras signaling pathway	226	12	5.69 × 10^–4^
Signal transduction	FoxO signaling pathway	134	9	0.001
Signal transduction	MAPK signaling pathway	253	12	0.001
Signal transduction	PI3K-Akt signaling pathway	345	14	0.001
Development and regeneration	Osteoclast differentiation	131	8	0.004
Sensory system	Inflammatory mediator regulation of TRP channels	98	7	0.004

KEGG, Kyoto encyclopedia of genes and genomes; FDR, false discovery rate

### Verification of molecular docking

**Fig. 5:**
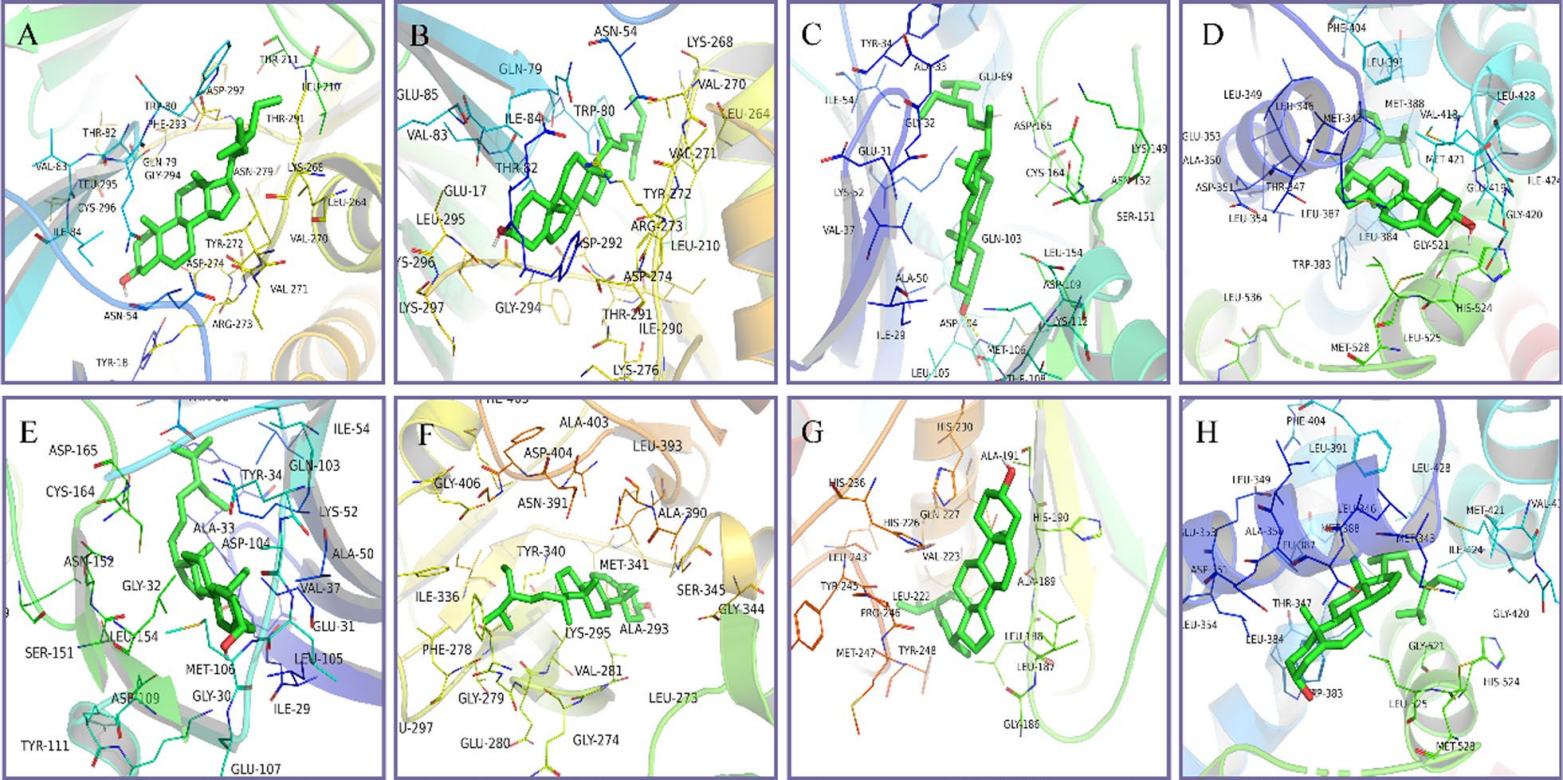
Molecular docking model diagram. **a** Molecular docking of AKT1 with stigmasterol. **b** Molecular docking of AKT1 with sitosterol. **c** Molecular docking of MAPK1 with stigmasterol. **d** Molecular docking of MAPK1 with sitosterol. **e** Molecular docking of ESR1 with stigmasterol. **f** Molecular docking of SRC with stigmasterol. **g** Molecular docking of MMP9 with stigmasterol. **h** Molecular docking of ESR1 with sitosterol

Molecular docking could effectively predict whether the ligand and the receptor could interact through the complementarity of the spatial structure and the principle of energy minimization in the region of the receptor active site [24, 25]. The lower the docking score between the ligand and the receptor, the greater the docking activity of the two and the more stable the structure. The molecular docking results showed that the molecular docking score between the active ingredients of RRP and the key targets was all less than − 4.2 kcal/mol, suggesting that these active ingredients have a certain affinity and binding activity with the key targets (Table 3). The docking score of the ligand and the receptor was less than − 7.0 kcal/mol, which indicated that they had strong binding activity. A total of 14 binding conformations have docking scores less than − 7.0. The top 8 binding relationships with the highest docking activity are AKT1–stigmasterol, AKT1–sitosterol, MAPK1–stigmasterol, ESR1–stigmasterol, MAPK1–sitosterol, SRC–stigmasterol, MMP9–stigmasterol, ESR1–sitosterol (Fig. 5).

**Table 3:** Docking score of the active ingredients of RRP and key targets

Molecule name	PDB ID	Docking score (kcal/mol)		
		Stigmasterol	Sitosterol	5-HMF
MAPK1	4s33	− 9.7	− 9.5	− 4.3
MAPK3	6ges	− 5.3	− 5.0	− 4.2
AKT1	6hhf	− 10.3	− 10	− 4.7
MAPK8	3pze	− 8.2	− 8.3	− 4.4
ESR1	2iok	− 9.6	− 8.7	− 4.3
PTGS2	4cox	− 7.9	− 8.1	− 5.0
EGFR	5y9t	− 6.3	− 5.9	− 3.8
FGF2	4fgf	− 5.0	− 4.6	− 3.2
SRC	4u5j	− 9.1	− 8.0	− 4.3
MMP9	6esm	− 8.9	− 8.6	− 5.6

### Bone densitometry results

**Fig. 6:**
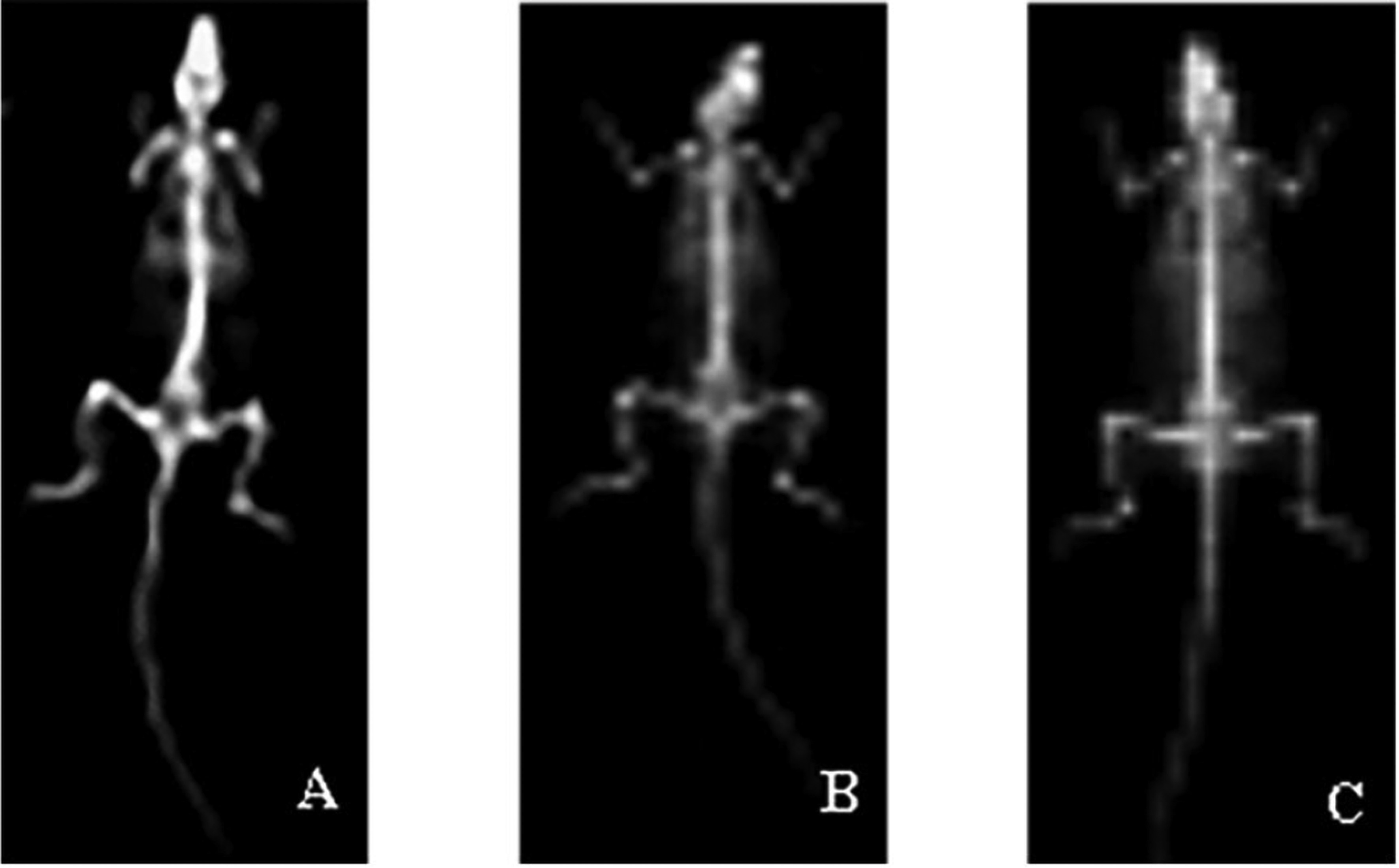
X-ray image of rat. **a** Sham operation group. **b** Model group. **c** RRP group

**Fig. 7:**
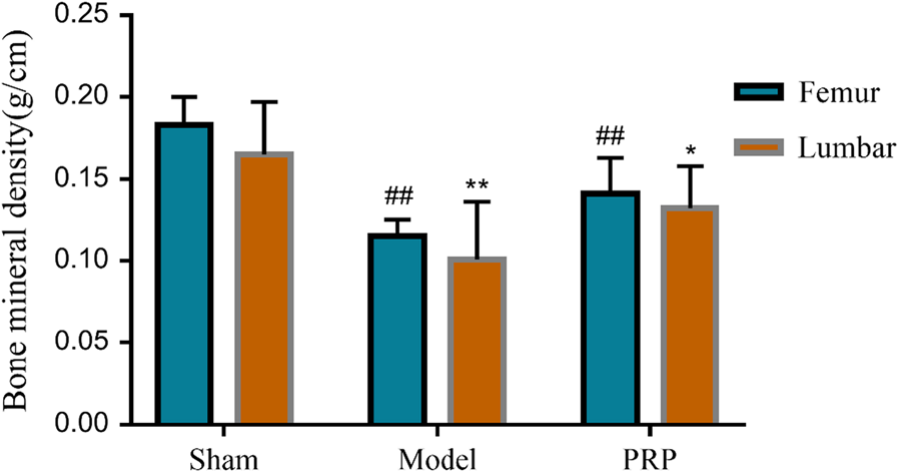
Effect on bone mineral density. Compared with the sham operation group, ^##^*P* < 0.01; compared with the model group, **P < 0.01, *P < 0.05

Compared with the sham operation group, the bone mineral density of the femur and vertebra in the model group was significantly decreased (*P* < 0.01). Compared with the model control group, the RRP group could significantly increase the bone density of the femur of ovariectomized rats (*P* < 0.01) and could significantly increase the bone density of the vertebral body (*P* < 0.05), as shown in Figs. 6 and 7.

### Effect on mRNAs of key targets

**Fig. 8:**
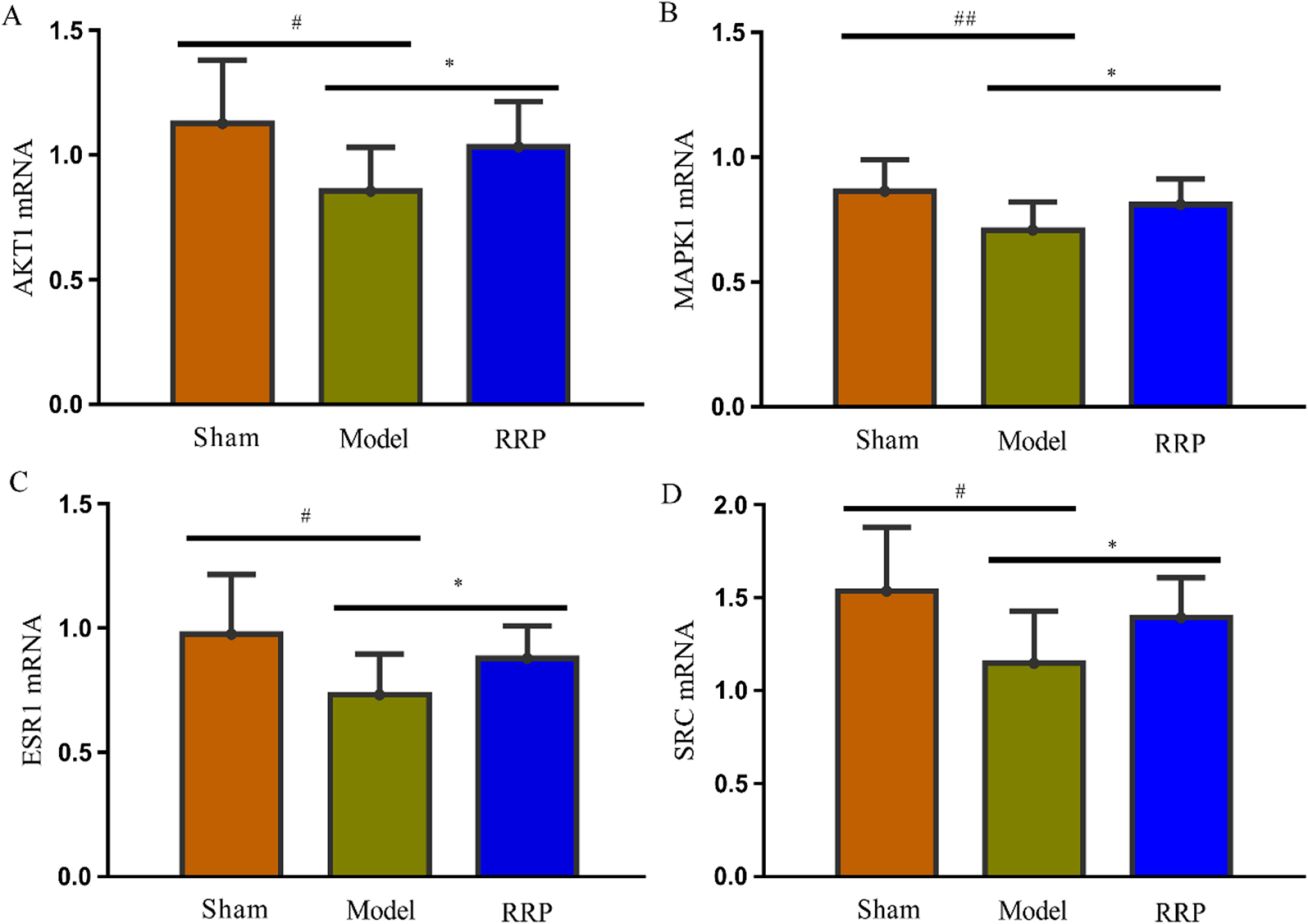
Effect on mRNAs of key targets. **a** Result of AKT1 mRNA expression. **b** Result of MAPK1 mRNA expression. **c** Result of ESR1 mRNA expression. **d** Result of SRC mRNA expression. Compared with the sham operation group, ^#^*P* < 0.05; compared with the model group, *P < 0.05

The relative quantitative expression levels of AKT1, MAPK1, ESR1, and SRC1 mRNA were calculated by the ^2−ΔΔ^CT method. The results showed that compared with the sham operation group, the expression levels of AKT1, MAPK1, ESR1, and SRC1 mRNA in the bone tissue of the model group decreased significantly (*P* < 0.05). Compared with the model group, the expression of AKT1, MAPK1, ESR1, and SRC mRNA in the bone tissue of the RRP group increased significantly (*P* < 0.05). The results showed that RRP could increase AKT1, MAPK1, ESR1, and SRC mRNA expression levels in the bone tissue of osteoporotic rats (Fig. 8).

## Discussion

The high morbidity and mortality of osteoporosis and osteoporotic fractures seriously affect the quality of life of the elderly and cause a huge economic and social health burden [26]. RRP can effectively prevent and treat osteoporosis [27], but the material basis and molecular mechanism of its treatment of osteoporosis are still unclear. A total of 76 active ingredients of RRP were searched, and three active ingredients were screened: β-sitosterol, stigmasterol, and 5-HMF. After removing the duplication, we obtained 428 RRP targets and 1179 targets related to osteoporosis, of which 118 overlapping targets were the common targets of RRP and osteoporosis. We used the Cytohub plug-in to analyze the PPI network topology properties. According to the node degree value, the top 10 genes are MAPK1, MAPK3, AKT1, MAPK8, ESR1, PTGS2, EGFR, FGF2, SRC, MMP9. The molecular docking results showed that the top four targets with the highest docking activity with the active ingredients of RRP were AKT1, MAPK1, ESR1, and SRC. Pathway enrichment analysis results showed that RRP treatment of osteoporosis was most closely related to the estrogen signaling pathway, and it is also associated with HIF-1, MAPK, and PI3K-Akt signaling pathways. These pathways belong to the endocrine system, signal transduction, developmental regeneration, and sensory system.

Estrogen is a critical regulatory hormone in the human body. Its physiological role is mainly accomplished by regulating the transcription and translation of target genes by acting on tissue cells' estrogen receptors (ESR). Bone tissue is a vital target tissue for estrogen. ESR1 and ESR2 are widely expressed in bone and bone marrow [28]. Studies have shown that estrogen plays a role in regulating bone metabolism mainly through its interaction with ESR1. After combining with ESR, estrogen controls the functions of osteoblasts and osteoclasts through various ways to participate in bone metabolism. It can promote the proliferation of osteoblasts, improve bone mineralization, and inhibit the activity of osteoclasts. In addition, estrogen can also affect bone metabolism through the calcium metabolism regulation system. In this study, the estrogen level in rats dropped rapidly after ovariectomy, and their bone density decreased significantly. A high-conversion osteoporosis animal model was successfully established.

AKT1 is a protein kinase that exists in osteoblasts and osteoclasts. It regulates cell proliferation and differentiation and is of great significance for maintaining bone mass. Activated PI3K phosphorylates the downstream signal protein Akt, which can reduce osteoclast autophagy [29]. The lack of AKT1 in the body will lead to the loss of bone mass, which will lead to osteoporosis [30]. In addition, AKT1 is a significant target for the treatment of avascular necrosis of the femoral head, which contributes to the repair of blood vessels after injury and the formation of articular cartilage blood vessels [31]. The HIF-1α signaling pathway plays a vital role in regulating the coupling process of bone formation and angiogenesis. VEGF is an important downstream gene of the HIF-1 signaling pathway, which can promote the formation of osteoclasts and increase the activity of osteoclasts [32, 33]. VEGF produced by mature osteoblasts is essential for angiogenesis–osteogenesis coupling [34, 35].

Activation of the MAPK pathway increases the proliferation and migration of osteoblasts, which can promote bone healing. Inhibition of MAPK signaling reduces the expression of specific genes in mature osteoblasts [36, 37]. Estrogen can promote the activation of MAPK/ERK signaling in osteoblasts. MAPK1 (ERK2) is the most abundant member of p38 in bone and bone marrow and plays a vital role in physiological bone homeostasis. ERK2 inhibitors can reduce the phosphorylation level of ERK2 and promote the adipogenic differentiation of human mesenchymal stem cells [38]. Adipocytes stimulate more hematopoietic stem cells to differentiate into osteoclasts, which leads to an imbalance in the coupling of osteoblasts to osteoclasts.

ESR1 can be expressed in osteoblasts, osteoclasts, chondrocytes, and bone marrow stromal cells and are closely related to many bone-related diseases [39–41]. ERK is an extracellular signal-regulated kinase that can regulate cell proliferation and differentiation. It is mainly activated by growth factors such as epidermal growth factor and insulin. Estradiol in chondrocytes inhibits phosphorylation and activation of ERK2 by targeting and activating ESR1, thereby increasing autophagic flux and reducing chondrocyte apoptosis. Studies have found that SRC1 gene knockout in female mice can significantly antagonize estrogen in promoting bone formation, indicating that SRC1 can positively regulate estrogen-related bone formation [42].

**Fig. 9:**
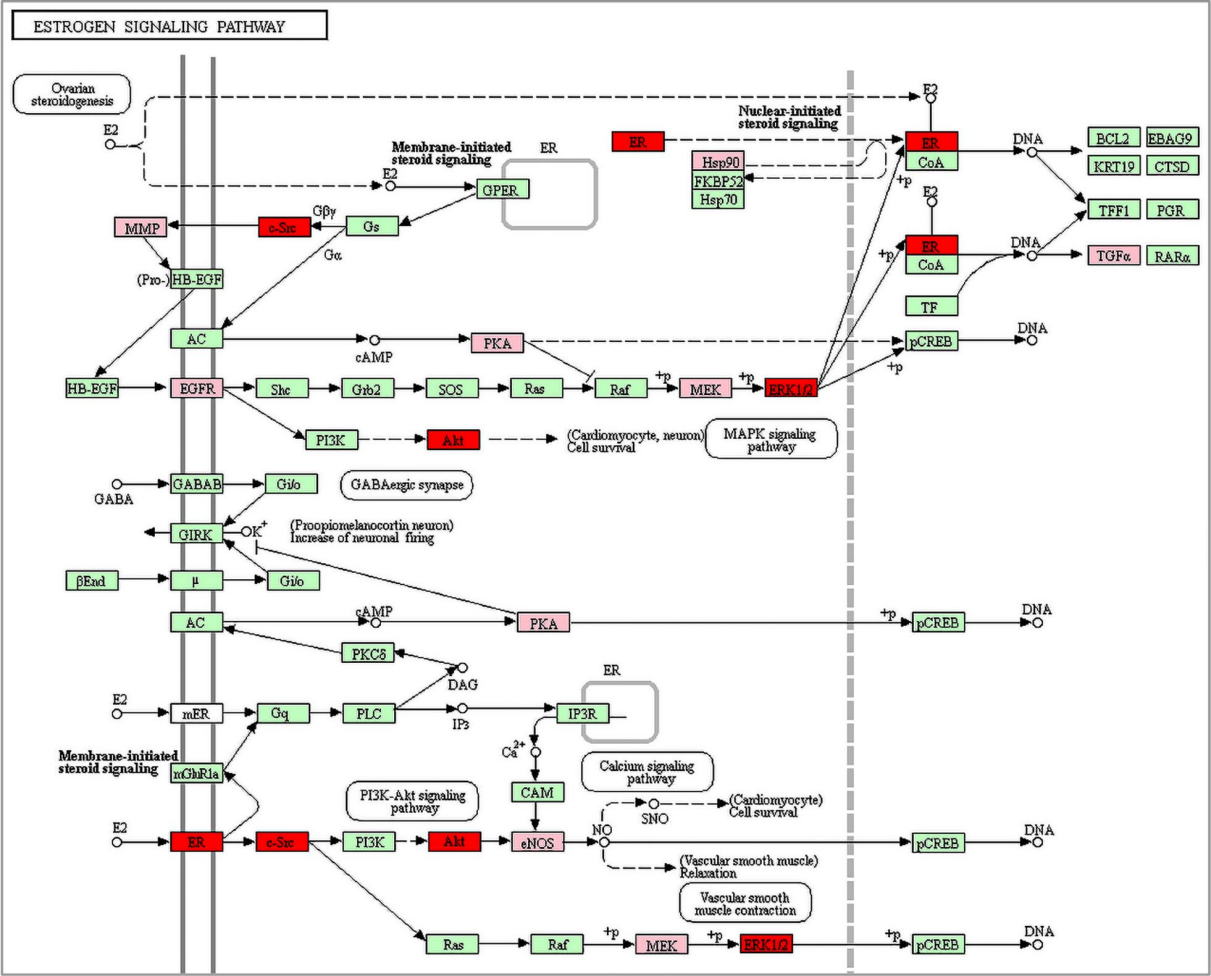
Estrogen signaling pathway (the pink nodes represent potential targets, the red nodes represent verified targets, the arrows represent the activation effect, and the T arrows represent the inhibition effect)

This research showed that RRP could significantly increase the relative expression of AKT1, MAPK1, ESR1, and SRC1 mRNA in the femur of ovariectomized rats. RRP might regulate the estrogen signaling pathway to prevent bone loss in ovariectomized rats (Fig. 9). At the same time, its role in preventing osteoporosis might also be related to the HIF-1 signaling pathway, MAPK signaling pathway, and PI3K-Akt signaling pathway.

## Conclusions

This study applied network pharmacology and molecular docking methods to study the material basis and potential mechanism of RRP in treating osteoporosis. RRP interferes with the biological process of osteoporosis through the endocrine system, signal transduction, development and regeneration, and sensory system. The experimental animal study showed that RRP could significantly increase Akt1, MAPK1, ESR1, and SRC1 mRNA expression levels in bone tissue to promote bone formation. This study explained the coordination between multiple components and multiple targets of RRP in the treatment of osteoporosis and provided new ideas for its clinical application and experimental research.

## Data Availability

All the data will be available upon motivated request to the corresponding author of the present paper.

## Abbreviations

RRP: *Rehmanniae Radix Preparata*
PPI: Protein–protein interaction
GO: Gene ontology
KEGG: Kyoto encyclopedia of genes and genomes
OB: Oral bioavailability
DL: Drug-likeness
RT-PCR: Real-time quantitative reverse transcription
FDR: False discovery rate
MAPK1: Mitogen-activated protein kinase 1
MAPK3: Mitogen-activated protein kinase 3
AKT1: Threonine protein kinase
MAPK8: Mitogen-activated protein kinase 8
ESR1: Estrogen receptor 1
PTGS2: Prostaglandin G
EGFR: Epidermal growth factor receptor
FGF2: Fibroblast growth factor 2
SRC: Proto-oncogene tyrosine protein kinase Src
MMP9: Matrix metalloproteinase-9
HIF-1: Hypoxia-inducible factor 1
VEGF: Vascular endothelial growth factor
TNF: Tumor necrosis factor
FoxO: Forkhead box protein O
PI3K: Phosphatidylinositol 3-kinase
